# Supplementary material for: MetaRibo-Seq measures translation in microbiomes
Source: Nat Commun. 2020 Jun 29;11:3268. doi: 10.1038/s41467-020-17081-z (PMC7324362; doi:10.1038/s41467-020-17081-z)
Supplement: Supplementary file 10 — Supplementary Data 7 [file 41467_2020_17081_MOESM10_ESM.zip › File2/Confidence_High_Taxonomy/30783_out.krona.html]

Javascript must be enabled to view this page.

members
magnitude
magnitudeUnassigned
count
unassigned
taxon
rank

30783\_out

3

3
superkingdom
2

2
phylum
1239

class
1
909932

order
1
909929

1843491
family
1

genus
1
970

671224
species

SRS023964\_contig\_number\_15830
1

1
class
91061

186826
order
1

family
1
1300

1301
genus
1

1
species

SRS147547\_contig\_number\_11033
1305

1
phylum
32066

203490
class
1

order
1
203491

1
family
203492

848
genus
1

species

SRS011306\_contig\_number\_contig-100\_19156.91921
1
860
